# Acute disseminated encephalomyelitis following the COVID-19 Vaccine Sinopharm in low- and middle-income country: a case report

**DOI:** 10.1097/MS9.0000000000001390

**Published:** 2023-10-06

**Authors:** Abhigan B. Shrestha, Mobin I. Mokbul, Tonmoy Chowdhury, Shubham Shrestha, Sajina Shrestha, Rupesh Raut, Md. Nuruzzaman

**Affiliations:** a M Abdur Rahim Medical College, Dinajpur; b Dhaka Medical College, Dhaka; c Rangamati Medical College, Bangladesh; d Patan Academy of Health Sciences, Lalitpur; e KIST Medical College, Imadol, Patan, Nepal

**Keywords:** acute disseminated encephalomyelitis, ADEM, COVID-19, severe acute respiratory syndrome coronavirus 2, Sinopharm, vaccine

## Abstract

**Introduction and importance:**

Coronavirus disease 2019 (COVID-19) is a disease caused by severe acute respiratory syndrome coronavirus 2 (SARS-CoV-2) and various vaccines against it have been developed. Acute disseminated encephalomyelitis (ADEM) is a disease of the central nervous system that cause inflammation and demyelination and manifests as a multi-symptom acute neurological condition. Although infections are usually the cause of ADEM, vaccines may cause 5–10% of cases.

**Case presentation:**

A 40-year-old woman had received a second dose of the Sinopharm COVID-19 vaccine 4 months before her visit and experienced sudden gait imbalance and vertigo a day after her vaccination, which lasted for more than a month. On examination, no signs of skin bruising or bleeding were observed, and her vital signs were within the normal range. On neurological assessment, the patient had a Glasgow Coma Scale score of 14/15 (E4V5M5), had normal pupil size and light reaction, normal fundus, normal deep tendon reflexes and bilateral extensor plantar response. Meningeal symptoms were absent, and SARS-CoV-2 RNA tests using NAAT (Nucleic Acid Amplification Test) were negative. Development of central nervous system (CNS) manifestations during the recovery phase of fever, along with typical MRI findings; the diagnosis of para-infectious ADEM with COVID-19 vaccination was made. After the treatment with methylprednisolone sodium succinate injection, the patient showed improvement.

**Clinical discussion:**

ADEM associated with post-vaccinations is a rare condition. There has been growing evidence that shared epitopes between neuronal proteins and SARS-CoV-2 antigens may trigger autoimmune reactions against the CNS through molecular mimicry as its pathogenesis.

**Conclusion:**

We suggest the need for a strict vaccine safety monitoring system and post-vaccine monitoring and surveillance.

## Introduction

Highlights

- Various vaccines against coronavirus disease 2019 (COVID-19) have been developed.
- COVID-19 vaccines can cause acute disseminated encephalomyelitis.
- We report a case of ADEM following the COVID-19 Vaccine Sinopharm.
- We suggest post-vaccine monitoring and surveillance.

Significant disruptions to the health, economic, and political systems were caused by the coronavirus disease 2019 (COVID-19) in 2020, but at the end of the year, optimism was born with the advent of a vaccine against the virus. Pandemic-ending COVID-19 vaccinations are now under development. Research into this virus’s mechanism has helped greatly in the development of vaccines and other preventative public health measures^1^.

Diseases of the central nervous system (CNS) that cause inflammation and demyelination include acute disseminated encephalomyelitis (ADEM). Clinically, it manifests as a multi-symptom acute neurological condition that may resolve in a single episode or progress to a chronic inflammatory central nervous system disorder^2^. Although infections are usually the cause of ADEM, vaccines may cause 5–10% of cases^2^. To date, few cases have been reported ADEM following COVID-19 vaccination. Using the Sinopharm vaccine, an inactivated virus COVID-19 vaccine, we describe a case of ADEM that manifested itself 2 weeks after the following dosage. Here we present a case of ADEM following the COVID-19 vaccine Sinopharm. This case has been reported following the SCARE (Surgical CAse REport) criteria^3^.

## Case report

A 40-year-old female had a history of vaccination for COVID-19 with a second dose 4 months prior to the visit. Then suddenly, after a day of vaccination, she developed an imbalance in gait and vertigo for more than a month. She was investigated by a primary care physician 2 days after the onset of symptoms and was prescribed drugs for her symptomatic management. With the symptoms persisting, the patient was admitted to our hospital after 4 months of vaccination.

Her general physical examination revealed an imbalance in gait and eyes closed most of the time. No petechiae, purpura, or ecchymotic lesions were seen and there was no evidence of mucosal bleeding. She was febrile (body temperature 99.6°F), although the temperature record showed an increasing pattern. Hemodynamic parameters were within normal limits. On neurological assessment, the patient had a Glasgow Coma Scale score 14/15(E4V5M5), had normal pupil size, as well as light reaction, normal fundus, normal deep tendon reflexes and bilateral extensor plantar response, pain and temperature sensation, proprioception, vibration sensation, and cranial nerve examination were normal. Meningeal signs were absent. Ear examination was normal and the patient had no history suggestive of peripheral causes of vertigo. The patient had no history of alcohol intake. The patient had no history of altered sensorium, personality changes, seizures, or any focal neurological deficits.

Investigation revealed hemoglobin of 10.9 g/dl, white blood cell count of 22.76 × 10^3^/μl (neutrophils 92.3%, lymphocytes 7.2%, basophils 0%, eosinophils 0%), platelet count of 284 × 10^3^/μl, erythrocyte sedimentation rate (ESR) of 25 mm/h, C-reactive protein (CRP) of 32 mg/l, blood urea of 46 mg/dl, serum creatinine of 1.4 mg/dl, blood glucose of 225.23 mg/dl, aspartate aminotransferase of (AST) 128 U/l, alanine aminotransferase (ALT) of 96 U/l, total bilirubin of 1.2 mg/dl, total protein of 5.7 g/dl, albumin of 3.2 g/dl, serum sodium of 132 mEq/l and serum potassium of 3.7 mEq/l. Cerebrospinal fluid (CSF) analysis showed 10 cells (all lymphocytes), mildly raised proteins (58 mg/dl) and normal glucose (68 mg%). Platelet count was monitored daily and values were reported to be within normal limits. Laboratory tests for the SARS-CoV-2 RNA using NAAT (Nucleic Acid Amplification Test) was negative. Peripheral smear for malaria parasite was negative and blood culture was sterile. Serological tests for human immune deficiency virus (HIV), hepatitis B and hepatitis C were negative.

**Figure 1:**
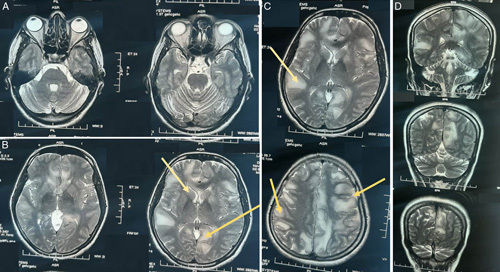
(A, B, C, D): T2-weighted MRI scan showing hyperintensities involving right temporal neocortex, bilateral symmetrical periventricular and subcortical white matter of frontal and parietal lobe.

Development of CNS manifestations during the recovery phase of fever along with typical MRI findings (Fig. 1A–D), the diagnosis of para-infectious ADEM with COVID-19 vaccination was made. The patient was treated with 1 g daily dose of methylprednisolone sodium succinate injection 1 vial with 200 ml normal saline for 5 days, ceftriaxone injection (1 g) bd (bis in die – twice a day), omeprazole injection (40 mg) bd, ondansetron injection (8 mg) tds (ter die sumendus – three times a day) was given. After slight improvement, the patient was discharged with prednisolone in tapering dose, vestibular sedatives, namely betahistine and prochlorperazine and antiemetic ondansetron. The patient showed further clinical improvement in the next 2 weeks of follow-up.

## Discussion

Acute disseminated encephalomyelitis (ADEM) associated with post-vaccinations or immunizations is a rare condition. The major criteria for ADEM are clinical attack of CNS demyelinating disease with acute or subacute onset, polysymptomatic neurologic features, and encephalopathy^4^. The diagnosis is done by taking into account the patient’s clinical history and symptoms along with supportive imaging findings, importantly, MRI sequences of T2-weighted and FLAIR (fluid-attenuated inversion recovery) images.

Multiple differential diagnoses were proposed initially based on our patient’s primary clinical presentation, such as Guillain Barre syndrome (GBS), multiple sclerosis (MS), autoimmune encephalitis, progressive multifocal leukoencephalopathy, toxic encephalopathies, etc. The clinical presentation of our patient did not support the diagnosis of GBS due to the presence of intact reflexes, the absence of sensory deficits, and abnormal brain MRI results. Similarly, the pattern of lesions seen in our patient’s MRI (i.e. the indistinct margin of the lesions and involvement of cortical and subcortical gray matters on both sides of the brain), no relapse of symptoms after steroid administration was in favor of the diagnosis of ADEM and were inconsistent with the diagnosis of MS. Autoimmune encephalitis is characterized by specific symptoms such as seizures, memory problems, and psychiatric symptoms, which were not observed in this case. Thus, our patient’s clinical signs of abnormal gait, vertigo, and positive MRI findings followed by the SARS-Cov-2 vaccine, rapid improvement after steroids and no further progression of neurologic deficits in follow-up or no relapse go in favor of vaccine-associated ADEM. The pathogenesis of post-vaccination ADEM is unknown, but there has been growing evidence that shared epitopes between neuronal proteins and SARS-CoV-2 antigens may trigger autoimmune reactions against the CNS through molecular mimicry^5^. As a result, antibodies cross-react with myelin autoantigens of neurons (e.g. myelin basic protein, myelin oligodendrocyte protein, proteolipid protein) in the CNS, resulting in the demyelination typically seen in ADEM. The second proposed mechanism suggests that ADEM may occur due to inflammation and increased vascular permeability in the CNS following vaccination or infection. This leads to mononuclear infiltration of the CNS vasculature, resulting in edema, hemorrhage, and damage to neuronal cells. The breakdown of the blood–brain barrier may also allow infiltration of the CNS by antigens and inflammatory cells, contributing to the cell-mediated immune response. ADEM can present with various clinical symptoms and prognoses due to the different degrees of damage caused by these mechanisms^6^. Post-COVID ADEM has been reported in almost all types of COVID vaccines such as AstraZeneca (ChAdOx1), Pfizer (BioNTech), Moderna, Sinopharm, Sputnik, Sinovac, Vero Cells, and Covaxin (BBV152). In our case, the vaccine was Sinopharm (China). There is a greater than expected occurrence of severe neurological adverse events such as cortical sinus venous thrombosis, Bell’s palsy, transverse myelitis, and GBSs along with other common effects such as headaches following different kinds of COVID-19 vaccination^7^.

It affects predominantly the ages between 30 and 50, and our patient was 40 years old, which is in line with previous data^8^. The mean time interval between vaccination and neurological symptoms was 14 days^5^. Immunosuppression is the mainstay of treatment for vaccine-associated ADEM. Patients are initially treated with steroids; plasmapheresis and intravenous immunoglobulin (IVIG) are reserved for those who do not respond to steroid therapy^8^. In our patient, we treated with prednisolone and methylprednisolone, and the patient responded excellently. Literature suggests that 85.1% of patients with ADEM after COVID vaccine had clinical improvements and only 13.8% of the patients died^5^. So, it is apparent that patients with ADEM typically have a good prognosis, with most patients having complete recovery or minor residual neurologic deficits^8^. Also, the incidence of post-vaccination ADEM remains low at about 0.1–0.2 per 100 000 vaccinated individuals^9^. Data from Vaccine Adverse Event Reporting System (VAERS) database shows the occurrence of ADEM following hepatitis, herpes papillomavirus (HPV), measles, mumps, and rubella (MMR), diphtheria, tetanus, and pertussis (DTAP), polio, and seasonal flu vaccines. However, vaccines against SARS-Cov-2, seasonal flu, and HPV were most frequently associated with ADEM, according to VAERS^10^.

## Conclusion

From a public health standpoint, though ADEM is a possible rare complication after SARS-Cov-2 vaccination, the benefits of vaccination outweigh the risks. We suggest the necessity of maintaining a strict vaccine safety monitoring system, post-vaccine monitoring and surveillance, immediate diagnosis and treatments after arising of any vaccine-associated complications. Nevertheless, we conclude that SARS-Cov-2 vaccination should not be ceased owing to the greater health interest of the mass people.

## Ethical approval

None.

## Consent

Written informed consent was obtained from the patient for publication of this case report. A copy of the written consent is available for review by the Editor-in-Chief of this journal on request.

## Sources of funding

No sources of funding.

## Author contribution

All authors have equally contributed to the writing, editing, and preparation of the manuscript and have reviewed it before submission.

## Conflicts of interest disclosure

There are no conflicts of interest.

## Research registration unique identifying number (UIN)

None.

## Guarantor

Abhigan Babu Shrestha.

## Data availability statement

None.

## Provenance and peer review

Not commissioned, externally peer-reviewed.
